# Electrofluids with Tailored Rheoelectrical Properties: Liquid Composites with Tunable Network Structures as Stretchable Conductors

**DOI:** 10.1021/acsami.4c07230

**Published:** 2024-08-08

**Authors:** Dominik S. Schmidt, Tobias Kraus, Lola González-García

**Affiliations:** † INM-Leibniz Institute for New Materials, Campus D2 2, 66123 Saarbrücken, Germany; ‡ Saarland University, Colloid and Interface Chemistry, Campus D2 2, 66123 Saarbrücken, Germany; § Saarland University, Department of Materials Science and Engineering, Campus D2 2, 66123 Saarbrücken, Germany

**Keywords:** carbon black, conductive networks, conductive composites, gauge factor, soft electronics, electrofluids

## Abstract

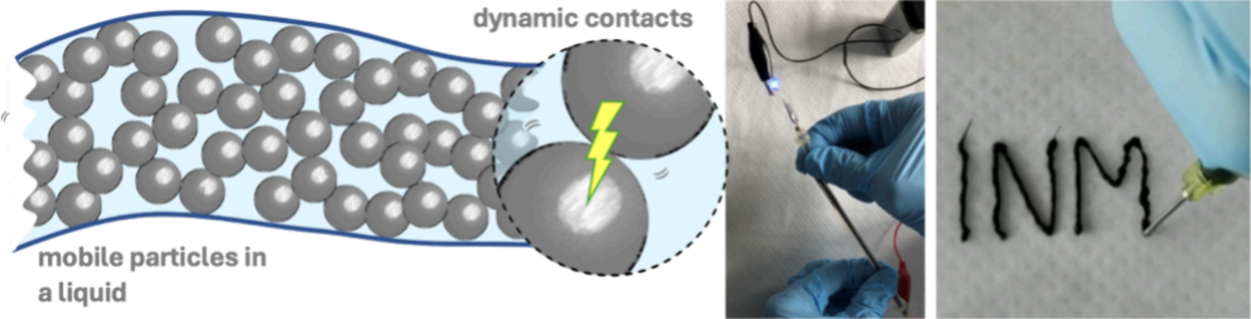

Flexible and stretchable electronics require both sensing elements and stretching-insensitive electrical connections. Conductive polymer composites and liquid metals are highly deformable but change their conductivity upon elongation and/or contain rare metals. Solid conductive composites are limited in mechanoelectrical properties and are often combined with macroscopic Kirigami structures, but their use is limited by geometrical restraints. Here, we introduce “Electrofluids”, concentrated conductive particle suspensions with transient particle contacts that flow under shear that bridge the gap between classic solid composites and liquid metals. We show how Carbon Black (CB) forms large agglomerates when using incompatible solvents that reduce the electrical percolation threshold by 1 order of magnitude compared to more compatible solvents, where CB is well-dispersed. We analyze the correlation between stiffness and electrical conductivity to create a figure of merit of first electrofluids. Sealed elastomeric tubes containing different types of electrofluids were characterized under uniaxial tensile strain, and their electrical resistance was monitored. We found a dependency of the piezoresistivity with the solvent compatibility. Electrofluids enable the rational design of sustainable soft electronics components by simple solvent choice and can be used both as sensor and electrode materials, as we demonstrate.

## Introduction

Soft electronic materials are from the basis of stretchable sensors,^1,2^ human-machine interfaces,^3^ wearables,^4,5^ and implants.^6^ Some are ideal conductors that retain their electrical conductivity under mechanical deformation; others are suitable as sensors because their electrical resistance reacts sensitively to pressure,^7^ tension,^7^ or bending.^8^ A common way to assess the sensitivity of a material toward deformation is the Gauge Factor (GF):
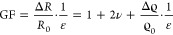
1

in which Δ*R* is the change in resistance, *R*_0_ the initial resistance, ε the strain, ν the Poisson’s ratio, Δϱ the change in resistivity, and ϱ_0_ the initial resistivity. The change in resistance with strain can be divided into the contributions of geometrical changes (based on Poisson’s ratio) and the change in intrinsic resistivity (piezoresistivity).

In sensor development, materials with large piezoresistivity are used to reach large GF. In classical semiconductor strain sensors, the contribution of the piezoresistivity exceeds the geometrical contribution by up to 70 times due to changes in their band structure,^9^ but they are susceptible toward mechanical fracture at small strains.^10^ Conductive polymer composites (CPCs) with conductive filler such as Carbon Black (CB),^11−14^ Carbon Nanotubes (CNTs),^15,16^ graphene,^17^ or metal particles^18,19^ in a nonconductive polymer matrix offer, in contrast, a large stretchability.^20^ Their gauge factors are dominated by the increasing particle distances under strain and/or crack formations and can be tuned; for example, Zheng et al. reported GF of 15.75 for CB in PDMS^21^ and Yang et al. up to 143 in graphene-silicone rubber nanocomposites.^22^

Connecting sensors while remaining overall stretchable requires low strain sensitivity conductors as electrodes. The yield strains of solid metals make it difficult to design such electrodes with them. Encapsulated liquid metals enable strains of up to 1000%^23^ but are not exempt of resistance changes due to geometry and exhibit GF similar to solid metals.^24^ Kirigami structures inspired by the traditional Japanese paper cutting technique have been used to create macroscopic structures that confer flexibility to rigid components^25^ and maintain low GF^26^ down to 0.25 as reported for Kirigami-structured graphene sheets at strains between 0 and 240%.^27^ Their macroscopic geometry requires tailored designs for each targeted application, complicating integration.

The conductive polymer composite community has also addressed this challenge using different strategies. For example, composites can be patterned to create wavy surfaces^28^ or serpentine circuits inspired in the above-mentioned Kirigami-structure to achieve ultrastretchable conductors that retain electrical properties at uniaxial strains over 300%.^29^ Another approach, sometimes combined with the previous one, is to use highly anisotropic conductive fillers such as silver nanowires or carbon nanotubes, which reduce the strain sensitivity of the composite in comparison to the use of spherical particles.^30−32^ Cai et al. combine single wall CNTs and a hydrogel matrix based on boric acid that utilizes self-healing properties to achieve gauge factors as low as 0.24 at 100% strain.^33^

A combined strategy uses liquid metal inclusions as conductive filler embedded in elastomeric matrices.^34,35^ Markvicka et al. and Tutika et al. demonstrated that the dispersion of liquid metal droplets into a PDMS matrix can yield stretchable connectors with minimal changes in resistance value upon deformation.^36,37^ Initially, the composite is electrically insulating since the liquid metal droplets are in contact through an oxide layer. Under local pressure, particles coalesce, forming a continuous conductive path. Upon mechanical deformation or severe damage, these composites have shown autonomous self-healing since the remaining discrete particles rupture and coalesce, reducing the change of the electrical resistance and achieving negligible GFs.

Silver flakes have been demonstrated to reduce the electrical sensitivity during strain since they can reconfigure and slide during the mechanical deformation, retaining the electrical conductivity of the filler network.^38,39^ Recently, Wang et al. reported an electronic interconnect based on silver flakes in a viscoelastic polymeric matrix.^40^ The liquid mixture was encapsulated in a stretchable elastomer. During stretching, silver flakes migrated toward the surface, densifying conductive paths and, therefore, increasing conductivity. The structural and electrical changes produced in the composite are, therefore, irreversible. As far as we know, this is the only example of a “liquid” composite reported as soft alternative electronic component; other particle types and the effect of different polymeric matrices remain unexplored.

Here, we demonstrate that microscopic percolating 3D networks of conductive particles can be formed in highly concentrated dispersions that we call “Electrofluids”. Their transient structures were tailored to tune their mechanoelectrical properties for stable conductors or sensors. Carbon Black (CB) particles were dispersed in solvents with different polarities and viscosities to create the electrofluids. The hierarchical CB with its aggregates of fused graphitic primary particles forms weakly bonded agglomerates in the liquid.^31^ The aggregates are stiff, but their contacts in the electrofluid agglomerates are transient, allowing electrical connections to constantly reform even when flowing. We show how the electrofluids’ hierarchical microstructure can be tuned by using solvents that lead to different particle–particle and particle–matrix interactions to design the electrofluids’ electromechanical properties. Their rheological and electrical responses under shear and uniaxial strain were investigated and the results were used to prepare conductors with tailored gauge factors. We demonstrate the use of electrofluids in soft robotics and wearables with a sensor-conductor prototype.

## Experimental Section

### Electrofluid Preparation

Used chemicals were acetylene black, 100% compressed (Alfa Aesar, Germany), PDMS Sylgard 184 (Dow Corning, USA), glycerol ReagentPlus ≥99% (SigmaAldrich, Germany), hexadecane ReagentPlus 99% (SigmaAldrich, Germany), hexane ReagentPlus 99% (SigmaAldrich, Germany), and ethylene glycol ReagentPlus ≥99% (SigmaAldrich, Germany). All chemicals were used as purchased without further purification. To prepare electrofluids, the desired amount of acetylene black powder was weighed in a vessel and covered with the liquid matrix. The mixing was done with a DAC 150.3 SP VAC-P speedmixer (Hauschild, Germany) at 2350 rpm for 3 min to obtain an electrofluid paste.

### Electron Microscopy

Scanning electron microscopy (SEM) images were recorded using a FEI Versa 3D DualBeam FIB-SEM system (FEI, USA) at 10 kV. Transmission electron microscopy (TEM) images were carried out with a JEM 2010 (JEOL, Germany) at 200 kV. To prepare the samples, the CB was dispersed in ethanol and treated in an ultrasonic bath. A droplet of the dispersion was placed on a silicon wafer (for SEM) or on a TEM grid and dried before the imaging.

### Raman Spectroscopy

The Raman measurements were performed with a Renishaw inVia Raman spectrometer at a wavelength of 532 nm. A small amount of sample was put onto a glass slide and put into focus. Wavelengths from 800 to 2200 cm^–1^ were tested.

### Electrical Conductivity

Conductivity measurements were done with a 4P-probe measurement to eliminate the wire and contact resistances. For that, the paste was filled in a custom-made, Teflon mold (1.5 × 1.5 cm^2^ with a thickness of 0.5 cm), which ensures applicability of the 4P-probe measurement model. The measurement was done using a Keithley Tectronix 2350 Sourcemeter (Tectronix, USA). A current–voltage curve was recorded, and the resistance was extracted from the slope of a linear fitting.

### Particle Size Characterization

The particle size distribution was analyzed using a LUMiSizer (LUM, Germany). The analysis of glycerol and PDMS samples was not possible due to the high viscosity of these solvents. Therefore, suspensions of 0.002 wt % CB in hexane and ethylene glycol were prepared and treated 20 min in an ultrasonic bath. Suspensions were then transferred into rectangular polyamide (PA) cuvettes. The centrifuge procedure used speeds between 2000 and 4000 rpm accumulating a total measurement time of 5 h. The analysis was done using StepView 6 software. The particle size distribution was determined using volume weighted analysis.

### Rheological Characterization

All rheological tests were performed with an Anton-Paar MCR302e rheometer. The measurements were done at 25 °C with a stainless-steel 25 mm parallel plate geometry with a gap height of 500 μm. Oscillatory rheology was performed in terms of an amplitude sweep with a strain from 0.01 to 100% with a fixed angular frequency of 10 rad/s. The LVE range was determined from the region of constant storage modulus. The yield point was determined as the strain, at which the storage modulus deviates 3% from the LVE region.

Rheoelectric measurements were done with coupling a Keysight E4980A dielectric spectrometer (Keysight, USA) to the rheometer. The temperature and geometrical conditions were the same as described above. The lower plate and the upper geometry served as electrodes for the electronic measurements. In oscillation mode, the same settings as described previously have been used. For each rheological measurement point, the electrical resistance was measured.

### Uniaxial Electromechanical Tests

All uniaxial electromechanical tests were performed on a ZwickRoell tensiometer (ZwickRoell, Germany). The test specimens were attached to the tensiometer and connected via copper wires to a Keithley Sourcemeter (see Figure S6 in the SI). For the cycling test, strain cycles of 10% were applied with a speed of 10%/s for a total of 2000 cycles. The first 1000 cycles were taken as initial equilibrium cycling until a constant signal was achieved. All further processed data was taken after this initial cycling. No waiting time was applied between the maximum and minimum strain load. For the different maximum strain values the sample was mounted the same way and was precycled for 1000 cycles at 10% strain. Afterward, different strain values from 10 to 100% were applied for 100 cycles at each strain amplitude. All strains were tested subsequently on the same sample, starting with the smallest deformation. After reaching 100% the strain was lowered from 75 to 10%. The change in resistance was recorded with a Keithley DAQ6510 Sourcemeter (Tectronix, USA).

### Demonstrator

Electrofluids were filled into silicone elastomer tubes and connected electrically via metal pins before sealing. One tube was filled with CB-PDMS 9 vol % and the other one with CB-Glycerol 9 vol %. Both tubes were attached to a glove and connected in series with a simple circuit containing a red and a blue LED that were controlled with an Arduino device. Illumination of the LEDs was triggered at different resistance values: blue for initial resistance and red for high resistance.

## Results and Discussion

**Figure 1:**
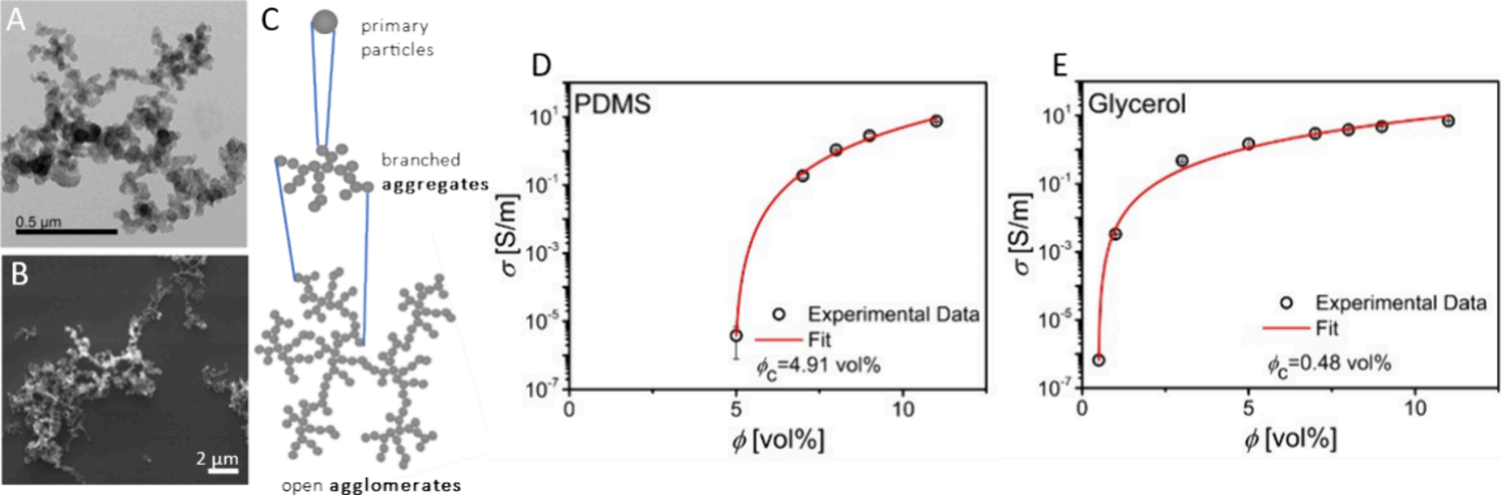
A: Transmission electron micrograph of Carbon Black (CB) aggregates and its constituent primary particles. B: Scanning electron micrograph of agglomerates formed in ethanol by weakly connected CB aggregates. C: Hierarchical structure of the CB filler. D: Percolation of the mixture CB-PDMS and E: Percolation of the mixture CB-glycerol. The red lines fit to a classical percolation relation described in eq 2 (more information can be found in Figure S2 in the SI).

Carbon Black (CB) is composed of primary particles that fuse into aggregates^41^ during production that are visible in transmission electron microscopy (TEM) (Figure 1A, see Experimental Section for details). In dispersion or during drying, weaker van der Waals forces connect the aggregates to agglomerates that are visible in scanning electron microscopy (SEM) (Figure 1B).^41^ The overall hierarchical structure of the CB that we used in this work is depicted in Figure 1C. Raman spectroscopy showed only characteristic carbon peaks at 1341 cm^–1^ (D-band) and at 1580 cm^–1^ (G-band),^42,43^ indicating the graphitic nature of the CB and the absence of functional groups on its surface (see Figure S1 in the SI). Such carbon surfaces are hydrophobic,^44^ suggesting that the powder will weakly interact with polar solvents and form stable dispersions in nonpolar liquids. The polarity of the solvent as well as its viscosity will affect CB agglomeration.

We prepared CB suspensions – “Electrofluids” – in liquid PDMS, hexadecane, glycerol, and ethylene glycol. Their electrical conductivities at different CB loadings were determined by measuring the electrical resistance in 4-point-probe configuration (details described in the Experimental Section). The percolation threshold (ϕ_c_) was then determined by fitting the electrical conductivity σ to
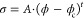
2

from classical percolation theory,^45^ where *A* is a constant, ϕ is volume fraction of the filler, and *t* is a critical exponent. The percolation thresholds strongly depended on the liquid matrix (Figures 1D,E and S2A,B in SI). Percolation in PDMS (Sylgard 184 base) occurred at nearly 5 vol % CB (Figure 1D). Silicone-CB mixtures have been extensively studied and are commonly used to create conductive solid composites by cross-linking the PDMS matrix.^46^ Carbon-based composites such as our carbon black electrofluid are generally less conductive than metal-based composites due to the lower intrinsic conductivities of carbon.^47^ The difference is amplified by the contact resistances between carbon particles (CB, CNTs, graphene flakes, etc.) that are generally higher than that of metal–metal contacts.^48^ However, they are of great interest for certain applications, in which electrical conductivity values are less critical, since they are sustainable (metal-free) and cost-effective.^49^ The percolation threshold and the conductivities that we found in suspensions are in good agreement with the values reported in the literature for the solid composites.^50−52^

All other solvents required less CB for percolation. Hexadecane is nonpolar like PDMS but has a lower viscosity (η_Hexadecane_ = 3.34 mPa·s versus η_PDMS_ = 4500 mPa·s) and caused a percolation threshold of 1.43 vol % CB, less than 1/3 of that in PDMS (see Figure S2A). A reduced percolation threshold for less viscous liquids has already been reported by Rwei et al.^53^ and attributed to a higher mobility of the CB that promotes filler reorganization and the formation of conductive pathways.^53^ Similar findings were reported by Bauhofer and Kovacs for carbon nanotubes.^54^

Glycerol is closer in viscosity to PDMS (η_Glycerol_ = 1480 mPa·s) but caused percolation at 0.48 vol % CB, 10 times below PDMS (Figure 1E). Glycerol is a small molecule with 3 OH-groups and a high polar moment, while the PDMS Si–O backbone is shielded by methyl groups that confer a hydrophobic character. The CB apparently forms large agglomerates that fill space with a connected network and reduce the percolation threshold in polar solvents.

Synergic effects of viscosity and polarity lent CB in ethylene glycol (η_Ethylene glycol_ = 16 mPa·s) the lowest percolation threshold of all studied systems (0.17 vol %, seeFigure S2B). Hexadecane has a similar viscosity but is nonpolar, which increased the threshold in comparison to ethylene glycol. The strong role of polarity on CB percolation that we find here has not been reported before. In the following, we will test our hypothesis that large agglomerates of CB aggregates form and strongly promote percolation.

**Figure 2:**
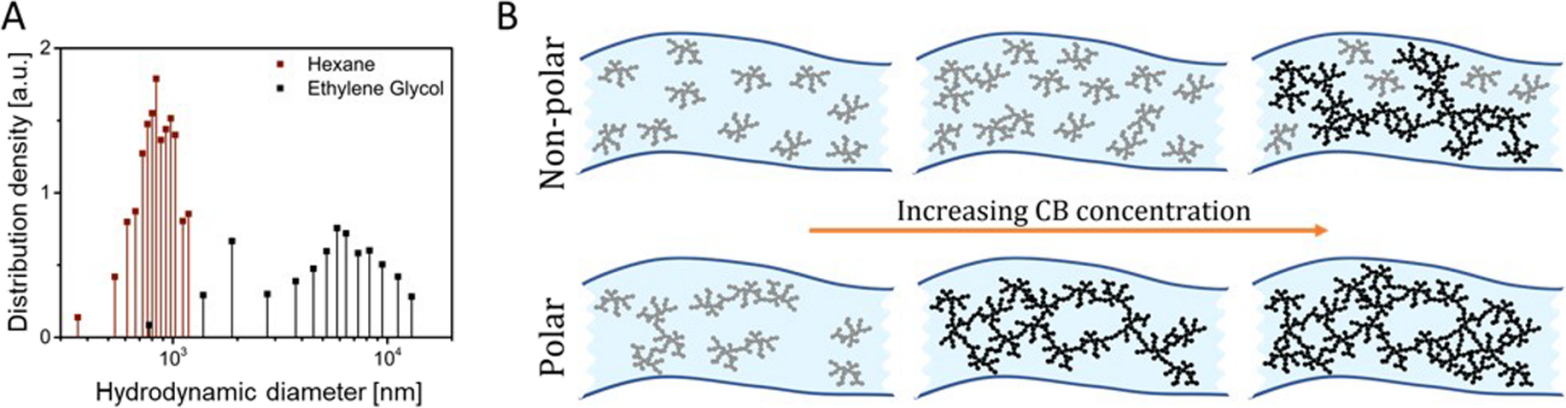
A: Size distribution densities of CB dispersed in ethylene glycol and in hexane. B: Proposed CB network formation in polar and nonpolar solvents.

We compared the sizes of CB agglomerates in nonpolar hexane and polar ethylene glycol using analytical centrifugation (AC) at relatively low CB concentrations of 0.002 wt %. Note that the highly viscous liquids PDMS and glycerol make AC cumbersome and imprecise (see Experimental Section for further details) and were not included. Figure 2A shows the size distributions with a mean hydrodynamic diameter in hexane of 840 nm. Ethylene glycol caused a bimodal distribution with maxima at 1942 and 6478 nm, indicating the formation of bigger agglomerates of CB. This result is in good agreement with Subramanian and Øye, who investigated the effect of lignosulfonates on CB dispersibility in water and found that the most hydrophobic lignosulfonates were the best dispersing agents and decreased the mean particle diameter by reducing agglomeration.^55^

Figure 2B illustrates the model that we propose for CB network formation based on above results. Electrofluids with low CB concentrations in nonpolar solvents contain CB aggregates and possibly small agglomerates with diameters of approximately 1 μm that disperse well in the matrix. The formation of networks at increasing CB concentrations in such solvents is dominated by the random percolation of CB units that collide.

Polar solvents cause stronger attractions between the CB, inducing the formation of larger agglomerates that are several μm in diameter. At very low concentrations, agglomerates cannot span the entire volume. As their concentration increases, they form a percolating network. Agglomeration facilitates percolation because larger agglomerates of CB are more efficient in filling space.

The percolation threshold in nonpolar solvents was lower than in polar ones of the same viscosity (cf. hexadecane and ethylene glycol in the SI Figure S2A,B). Their percolation thresholds were smaller than those of more viscous solvents of the same polarities because their reduced viscosity facilitates the formation of agglomerates.^53^

**Figure 3:**
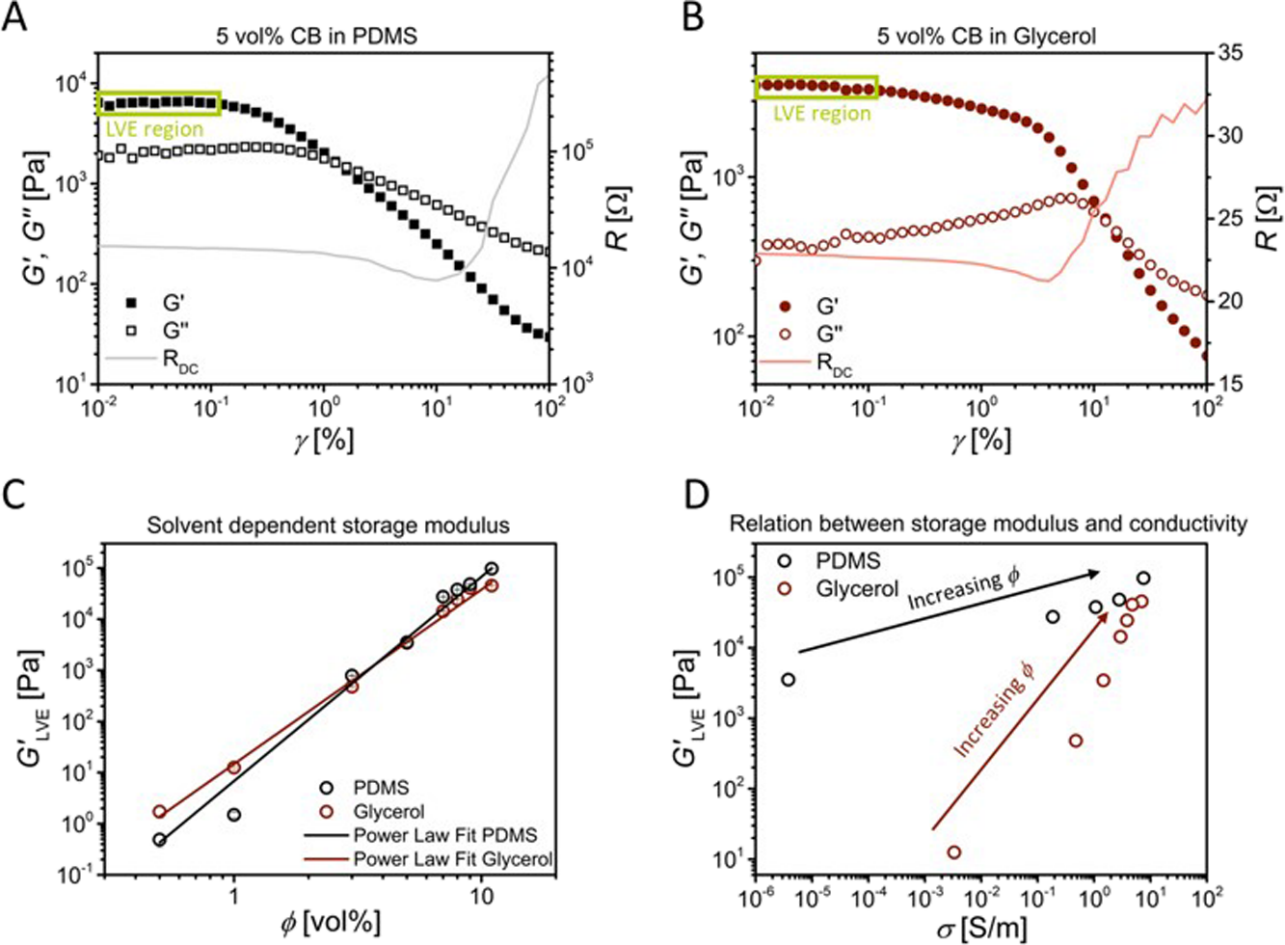
Oscillatory amplitude sweeps at ω = 10 rads^–1^ in the range of 0.01–100% strain. Closed symbols: Storage Modulus. Open symbols: Loss Modulus. Light line: Resistance. A: 5 vol % CB in PDMS. B: 5 vol % CB in Glycerol. C: Storage Modulus as a function of filler volume fraction and used solvent. Experimental points were fitted with a power law *G′* ∼ ϕ^*n*^. D: Storage Modulus’ plateaus as a function of conductivity. Volume fractions were 5, 7, 8, 9, and 11 vol % for PDMS and 1, 3, 5, 7, 8, 9, and 11 vol % for glycerol.

Electrofluids combine electrical conductivity with mechanical adaptability. The electrical percolation study presented above confirms the formation of electrically conductive filler networks and the role of solvent polarity on its formation. These results do not provide, however, insight into the mechanical properties of the formed networks, and mechanical and electrical properties of networks do not necessarily overlap.^56^ Therefore, we studied the viscoelastic behavior of the electrofluids and characterized the storage modulus *G′* that represents the elastic stored energy and accounts for the stiffness of the material and the loss modulus *G′′* that represents the dissipated energy and, therefore, the damping of the material by means of oscillatory rheology. Electrodes were coupled to the rheometer (see Experimental Section for details) to concurrently measure the electrical resistance of the samples at each strain in situ. The results for electrofluids containing 5 vol % CB in PDMS and glycerol are shown in Figure 3A,B (cf. Figure S2A,B in the SI for hexadecane and ethylene glycol).

The storage modulus *G′* was, in all cases, larger than the loss modulus *G′′*, indicating the presence of a mechanical network. The shape of the curves resembles the typical gel-like behavior, in which the *G′* remains constant and over the *G′′* at low strains (linear viscoelastic (LVE)-region), followed by a decrease (yield point, γ_y_ or τ_y_), and a crossover between the storage and the loss moduli (*G′* = *G′′*, flow point, γ_f_ or τ_f_) at increasing strains, indicating the transition from a solid-like to a liquid-like behavior.^57^

We focus now on electrofluids containing 5 vol % CB in PDMS and in glycerol, i.e., Figure 3A,B. Their *G′* were found to be on the same order of magnitude (6390 Pa for CB-PDMS and 3760 Pa for CB-glycerol), suggesting slightly higher stiffness of CB-PDMS, even when the proximity to the percolation threshold is 1 order of magnitude different (ratio ϕ/ϕ_c_ of 1.02 for CB-PDMS and 10.42 for CB-glycerol). The brittleness of the systems, however, differed considerably. A way of estimating it is the so-called flow transition index (FTI), which is defined as the ratio between the stress at the flow point and at the yield point τ_f_/τ_y_. The closer the ratio is to 1, the higher is the tendency of the sample to undergo brittle fracture. In this context, it means that the initial network structure is broken or unable to resist the deformation and, therefore, starts flowing. We found the CB-PDMS system to be more brittle (FTI = 5.3) than the CB-glycerol (FTI = 51.7) at 5 vol % of CB. This is consistent with the model described above, CB aggregates are well-dispersed in PDMS and the network in PDMS at 5 vol % contains only few CB-CB contacts. When they break at the yield point, the network cannot take up force anymore, and the electrofluid flows almost immediately. The CB network in glycerol at 5 vol % is mechanically more stable because more CB-CB contacts exist (electrical percolation found at 0.41 vol % CB), and CB-CB interactions are stronger. At the yield point, some CB-CB contacts in the network break, but the network is strong enough to sustain plastic deformations without flowing. The peak in *G′′* at ∼6% strain in Figure 3B indicates the breakup of the CB mechanical network; its area is known to be correlated with the energy dissipated in the process.^57^

Relations between mechanical and electrical networks were investigated with in situ resistance measurements during the rheological tests (see Experimental Section for details). Figure 3A,B also shows the evolution of the electrical resistance (*R*_DC_) during the oscillatory test. The evolution of their resistances with strain were in line with the different rheological regions described above. At low strain, in the LVE-region, the signal remained almost constant since the mechanical network absorbed the deformation elastically. The overall resistances were 15,300 and 23 Ω for CB-PDMS and CB-glycerol, respectively, a large difference that is in good agreement with the values of electrical conductivity presented in the corresponding percolation curves shown in Figure 1D,E. At the strain where *G′′* increased and plastic deformations ensued, the filler network was destroyed, and electrical resistances increased. The materials retained electrical conductivity even far beyond the flow point. We argue that this is due to transient contacts in a “steady state”, where network connections form and break at identical rates. This transient network is electrically conductive but mechanically weak.

Electrofluids based on 5 vol % of CB in hexadecane and ethylene glycol (Figure S3A,B in SI) were sufficiently far above the electrical percolation threshold to exhibit low electrical resistances at rest and were rheologically similar. Both presented comparable evolutions of *G′* and *G′′* and the changes of their resistances with strain were in line with the different rheological regions described above. At low strains, resistance remained constant, because the mechanical network absorbed the deformation elastically. At the strain where *G′′* increased, the electrical resistance increased as well until reaching a plateau at strains larger than the flow point that we attribute to the transient CB network introduced above.

The overall stiffness of all electrofluids, represented by the *G′* value at the LVE-region, was dominated by the filler–filler and filler–matrix interactions. Figure 3C shows that the values of the storage modulus (LVE-region from amplitude sweeps) scaled with CB volume fraction as *G*^′^ ∼ ϕ^*n*^, with *n* = 3.99 for the nonpolar PDMS and *n* = 3.41 for the polar glycerol.

Glycerol interacts weakly with the nonpolar CB that formed a mechanically strong network below 1 vol % CB. The storage modulus increased with CB loading because the network became mechanically stronger. The dispersed CB aggregates in PDMS-based electrofluids (Figure 2B) interact with the polymer chains and reduce their mobility, reinforcing the mixture even at CB loadings below percolation. This effect is weaker than that of agglomeration in glycerol-based electrofluids, which was reflected in the values of the storage moduli. Similar relations between filler dispersion and the stiffness of solid composites have been reported elsewhere.^58^ At CB contents above percolation (5 vol %) in PDMS, a mechanically rigid network formed. The combined contributions of the filler–matrix interactions and the mechanical network formation explain the higher scaling exponent for PDMS than for glycerol.

A low elastic modulus at a high electrical conductivity is beneficial for soft conductors that can be formed by filling electrofluids in an elastic tube, for example. Figure 3D gives the relation between the plateau *G′* values and the electrical conductivity (σ) of the resting electrofluid. The increase of conductivity with *G′* suggests a direct correlation with the stiffness, but the exact relation depends on the polarity of the solvent. The scaling of *G′* in glycerol-based electrofluids followed a power law, while that in PDMS only for volume fractions above 7 vol % CB. In consequence, glycerol-based electrofluids provide higher conductivities at comparable stiffness. These results highlight the close interplay between the electrical and the mechanical networks and thus, the macroscopic material properties. The nonlinear relations enable rational tuning of the rheoelectrical properties of electrofluids.

**Figure 4:**
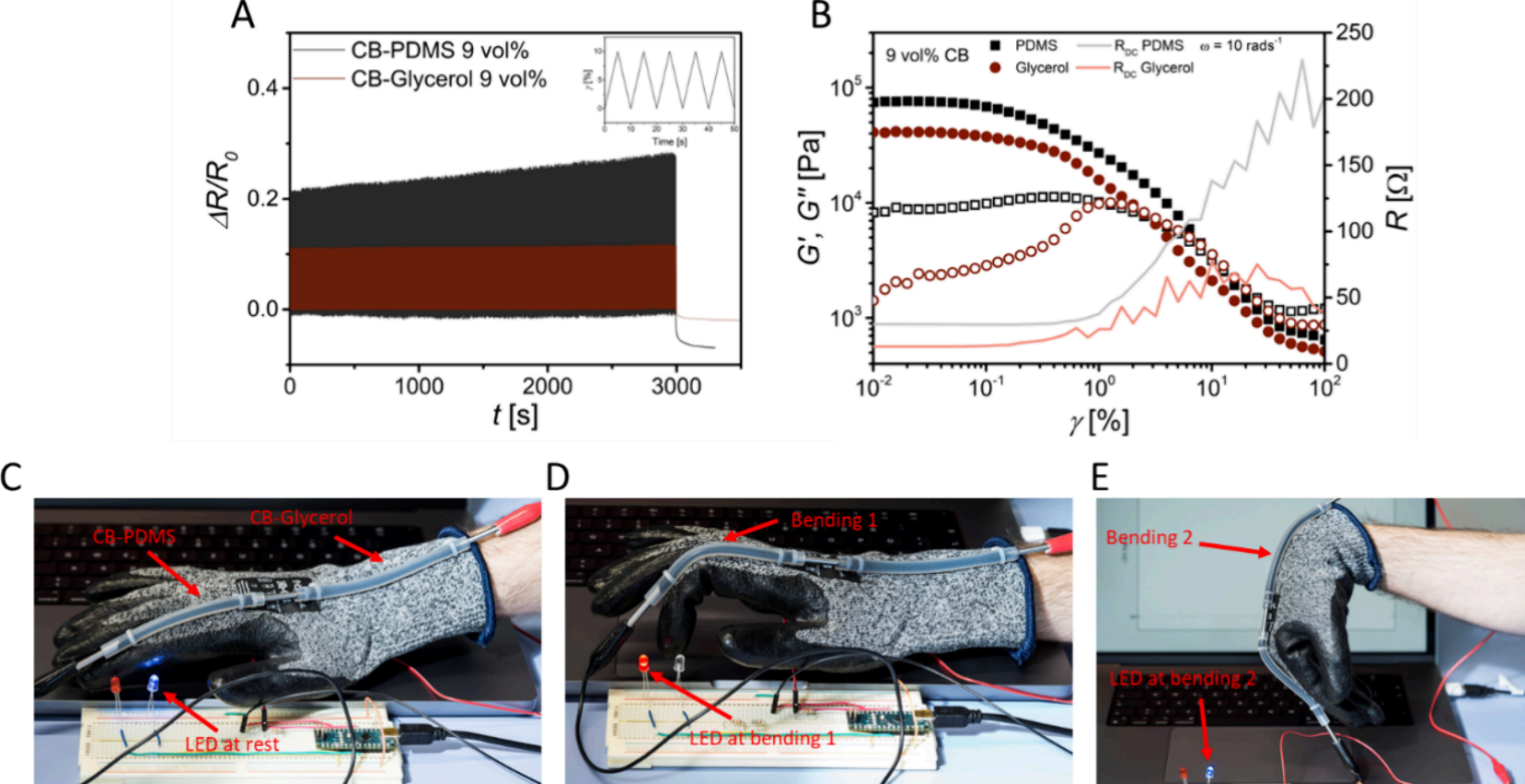
A: Change in resistances measured during cyclical loading between 0 and 10% strain (1000 cycles) for 9 vol % CB in glycerol and PDMS. The inset shows the strain profile. B: Rheoelectrical characterization of 9 vol % CB in PDMS and glycerol. C–E: Electrofluid-filled tubes attached to a glove. A sensing electrofluid senses the motion of the finger region; it is connected to a permanently conductive electrofluid lead at the wrist. A blue LED lights at low resistance of the connected tubes, a red LED indicates high resistance. C: Sensing glove at rest. D: Bending the finger triggers the red LED. E: Bending the wrist does not change resistance. A video of the demonstrator operation can be found as SI.

Consider the use of electrofluids in soft robotics. Some components of soft robots require stable electrical conductivity upon mechanical deformations, others (sensors) must have a high sensitivity to detect small pressures, compressions, strains, bending, etc. We tested the suitability of electrofluids for both cases and loaded different types in silicone tubes that were electrically connected at their ends (see details in the Experimental Section). We quantitatively evaluated the electrical response of encapsulated electrofluids under uniaxial strain and refer to their “piezoresistivity” in the following, extending the term that is well-established in conductive composites analogously for electrofluids. Uniaxial tensile tests were performed on electrofluids with 9 vol % CB in PDMS and in glycerol, and the electrical resistance changes were measured in situ. The CB concentrations ensured percolation and good electrical conductivity in both solvents. We subjected the sample to cyclical loads resulting in 0–10% strain at a constant strain rate of 10% per second (Figure 4A). Electrical resistance was recorded during the entire experiment. All details on the setup can be found in the Experimental Section and in Figure S4 in the SI.

Figure 4A shows the relative changes in electrical resistances during 1000 cycles. The electrical resistance change for the PDMS-based electrofluid was much higher than that for the glycerol-based one. A common parameter that is suitable to evaluate this difference is the gauge factor (GF, see eq 1 in the Introduction). The gauge factor of the 9 vol % CB-glycerol was 1.02 ± 0.01, that of 9 vol % CB-PDMS was 2.55 ± 0.2, i.e., more than double, indicating a higher sensitivity. This difference is in good agreement with the corresponding rheoelectrical measurement (Figure 4B), where the increase of electrical resistance beyond the LVE-region was larger for PDMS than for glycerol. Rheoelectrical measurements thus qualitatively predicted the uniaxial tensile test results for electrofluid-based devices and enabled a rational fluid design for specific applications.

When a solid conductive composite sample is stretched, its overall resistance tends to increase. This increase can have a geometrical and a piezoresistive component. A material with a Poisson’s ratio of 0.5 (incompressible material, e.g., rubbers and their composites) with a GF = 2 does not exhibit piezoresistivity, i.e., the resistivity of the material remains constant and the change in resistance is purely due to its geometrical deformation. A GF > 2 indicates positive piezoresistivity, i.e., an increase of the material’s resistivity upon strain. Our electrofluids based on CB-PDMS had GF > 2, and their response can be compared to that of liquid metals in tubes, which typically have gauge factors around 2.^24^ A GF < 2 indicates negative piezoresistivity; such materials are poor sensors but stable conductors. Our glycerol-based electrofluid falls in this category, indicating a change in the CB network that decreases the intrinsic electrical resistivity of the electrofluid. Even small changes in the particle contact, i.e., closer contact or increased contact area, are sufficient to improve the electrical conductivity of the overall network and compensate for the geometrical changes.

The difference between the electrofluids’ GF can be explained when considering CB-solvent interactions. PDMS is a nonpolar polymer that interacts strongly with CB aggregates. In solid composites, positive piezoresistivity is typically attributed to the particles losing contact forced by the alignment of the polymer chains during stretching^38^ and the formation of cracks;^59^ the second last cause is not applicable in the case of electrofluids. In the electrofluids, CB aggregates interact with the PDMS polymer chains as was concluded from the rheological measurements (see section above). The strongly adsorbed polymers reduce CB-CB interactions and make the joints weaker. Upon stretching, the polymer chains align, and the CB network gets disrupted, losing some of the connections.

Glycerol is, however, a small polar molecule that poorly interacts with the CB aggregates. Upon stretching, the large agglomerates are pulled apart, but due to the poor solvent affinity they will reconfigure to maximize CB-CB contacts. A large part of the geometrical changes is thus compensated, leading to a GF smaller than 2.

We exploited the solvent-dependent differences in the electrofluids properties to design electromechanical components. Figure 4C–E shows elastomer tubes filled with 9 vol % CB in PDMS and 9 vol % CB in glycerol, respectively (see Video 1 as electronic SI). We connected them in series and attached them to a glove. At rest, the electrical signal was constant, and the blue LED was on. Bending the finger increased the resistance of the CB-PDMS electrofluid, triggering the red LED. Bending the wrist deformed the CB-glycerol electrofluid, but its resistance increase was small, and the red LED remained off. A combination of the two electrofluids thus enabled the design of robotic structures, in which one part serves as a deformation sensor while the other faithfully transmits the signal.

**Figure 5:**
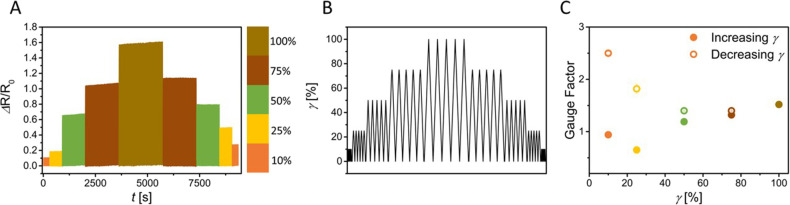
A: Change in resistance of a 9 vol % CB in glycerol sample stretched to different strain amplitudes. From orange to brown 100, 75, 50, 25, and 10%. Each strain value was cycled for 100 cycles. B: Used Strain profile for the electromechanical test. C: Gauge factors of 9 vol % CB-glycerol electrofluid at the different strain values. Filled symbols indicate increasing strain amplitude and open symbols decreasing strain amplitude.

To assess the limits of the electrofluid containing 9 vol % CB in glycerol as stable signal transmitter, we investigated the relation between uniaxial deformation and electrical response at larger uniaxial strains. A silicone tube containing the electrofluid underwent 100 cycles at increasing and decreasing strain (10%–100%–10%) at a constant rate, while monitoring the electrical resistance changes (see Experimental Section for details). The results are summarized in Figure 5. At increasing strain, the changes in electrical resistance also increased; however, when calculating the GF, which considers the geometrical changes and the strain, we observed that all the values were below 2, indicating a decrease in resistivity that opposes the mechanical deformation. Interestingly, when the electrofluid underwent lower strain cycles (75–10%) after the maximum strain (100%), the changes in resistance were larger compared to the initials, particularly marked at low strains, resulting in a GF larger than 2 at 10% strain. This could be an indication of strain memory of the material. For example, if this electrofluid undergoes small strains during application, a GF = 1 at 10% strain is to be expected; if, however, undergoes large deformations, the GF at 10% strain afterward is expected to be much larger, 2.5 (see Figure 5C).

## Conclusions

In this work, we introduced “Electrofluids” as electrically conductive liquid composites and demonstrated their tunability as flexible, stretchable conductors and as strain sensing materials. Carbon Black (CB) was used as an electrically conductive filler. Suspensions with increasing CB concentration in polar and nonpolar solvents exhibited large differences in mechanical network strength and electrical conductivity. We explained the differences using a model based on the affinity of the conductive filler toward the liquid matrix. High affinity toward nonpolar liquids led to smaller agglomerates and increased both the percolation threshold and the stiffness at higher volume fractions. Low filler-solvent affinities in polar liquids led to larger agglomerates even at low concentrations, reducing the percolation threshold. In situ rheoelectrical measurements showed a close connection between the mechanical and the electrical network in the systems, giving a direct correlation between the stiffness and electrical conductivity. Polar solvents delivered an improved figure of merit, i.e., higher conductivity at comparable stiffness.

We tested the sensitivity of different electrofluids’ conductivity to uniaxial strain and found that electrofluids with nonpolar liquids present slight piezoresistivity and higher electrical sensitivity toward uniaxial strains. Surprisingly, electrofluids based on polar liquids retained better the electrical conductivity even at high strains (100%) since the CB network reconfigures and compensates the geometrical changes imposed by the strain. Both exhibited good reproducibility of their responses over cycles. We used a demonstrator to show that electrofluids with the same filler work as strain sensor and a stable conductor. This single-component strategy reduces production costs and enables more sustainable materials simplifying the recyclability processes.

We envision the use of fillers and liquids that expand the electrofluid portfolio to achieve a wide range of electromechanical properties. Metal particles, flakes, and wires, or their mixtures, with liquids should lead to a broad range of electromechanical responses. For example, 1D objects such as carbon nanotubes reduce the percolation threshold and can align under suitable flow conditions, imparting memory to the electrofluids.

## Supporting Information Available

Additional experimental details (Raman spectra, electrical percolation, rheoelectric measurements) and images of experimental setup (PDF)
Electrofluids: strechable sensors and leads; carbon black in PDMS and glycerol; and increasing resistance due to bending (MP4)
Application of electrofluids in a demonstrator (MP4)

## References

[ref1] Ma L.; Shuai X.; Hu Y.; Liang X.; Zhu P.; Sun R.; Wong C.-P. A Highly Sensitive and Flexible Capacitive Pressure Sensor Based on a Micro-Arrayed Polydimethylsiloxane Dielectric Layer. Journal of Materials Chemistry C 2018, 6 (48), 13232–13240. 10.1039/C8TC04297G.

[ref2] Wang Z.; Gao W.; Zhang Q.; Zheng K.; Xu J.; Xu W.; Shang E.; Jiang J.; Zhang J.; Liu Y. 3D-Printed Graphene/Polydimethylsiloxane Composites for Stretchable and Strain-Insensitive Temperature Sensors. ACS Appl. Mater. Interfaces 2019, 11 (1), 1344–1352. 10.1021/acsami.8b16139. 30523681

[ref3] Chen T.; Shi Q.; Zhu M.; He T.; Sun L.; Yang L.; Lee C. Triboelectric Self-Powered Wearable Flexible Patch as 3D Motion Control Interface for Robotic Manipulator. ACS Nano 2018, 12 (11), 11561–11571. 10.1021/acsnano.8b06747. 30335958

[ref4] Hwang B.-U.; Lee J.-H.; Trung T. Q.; Roh E.; Kim D.-I.; Kim S.-W.; Lee N.-E. Transparent Stretchable Self-Powered Patchable Sensor Platform with Ultrasensitive Recognition of Human Activities. ACS Nano 2015, 9 (9), 8801–8810. 10.1021/acsnano.5b01835. 26277994

[ref5] Meng K.; Wu Y.; He Q.; Zhou Z.; Wang X.; Zhang G.; Fan W.; Liu J.; Yang J. Ultrasensitive Fingertip-Contacted Pressure Sensors To Enable Continuous Measurement of Epidermal Pulse Waves on Ubiquitous Object Surfaces. ACS Appl. Mater. Interfaces 2019, 11 (50), 46399–46407. 10.1021/acsami.9b12747. 31814402

[ref6] Melzer M.; Makarov D.; Schmidt O. A Review on Stretchable Magnetic Field Sensorics. J. Phys. D: Appl. Phys. 2020, 53 (8), 083002 10.1088/1361-6463/ab52cf.

[ref7] Chen J.; Zhu Y.; Chang X.; Pan D.; Song G.; Guo Z.; Naik N. Recent Progress in Essential Functions of Soft Electronic Skin. Adv. Funct. Mater. 2021, 31 (42), 2104686 10.1002/adfm.202104686.

[ref8] Rao Z.; Ershad F.; Almasri A.; Gonzalez L.; Wu X.; Yu C. Soft Electronics for the Skin: from Health Monitors to Human–Machine Interfaces. Adv. Mater. Technol. 2020, 5 (9), 2000233 10.1002/admt.202000233.

[ref9] Barlian A. A.; Park W.-T.; Mallon J. R.; Rastegar A. J.; Pruitt B. L. Semiconductor Piezoresistance for Microsystems. Proceedings of the IEEE 2009, 97 (3), 513–552. 10.1109/JPROC.2009.2013612. 20198118 PMC2829857

[ref10] Zhang S.; Cai L.; Li W.; Miao J.; Wang T.; Yeom J.; Sepúlveda N.; Wang C. Fully Printed Silver-Nanoparticle-Based Strain Gauges with Record High Sensitivity. Adv. Electron. Mater. 2017, 3 (7), 1700067 10.1002/aelm.201700067.

[ref11] Karuthedath C. B.; Fikri U.; Ruf F.; Schwesinger N. Characterization of Carbon Black Filled PDMS-Composite Membranes for Sensor Applications. Key Engineering Materials 2017, 753, 18–27. 10.4028/www.scientific.net/KEM.753.18.

[ref12] Shintake J.; Piskarev E.; Jeong S. H.; Floreano D. Ultrastretchable Strain Sensors Using Carbon Black-Filled Elastomer Composites and Comparison of Capacitive Versus Resistive Sensors. Adv. Mater. Technol. 2018, 3 (3), 1700284 10.1002/admt.201700284.

[ref13] Zhai W.; Xia Q.; Zhou K.; Yue X.; Ren M.; Zheng G.; Dai K.; Liu C.; Shen C. Multifunctional Flexible Carbon Black/Polydimethylsiloxane Piezoresistive Sensor with Ultrahigh Linear Range, Excellent Durability and Oil/Water Separation Capability. Chemical Engineering Journal 2019, 372, 373–382. 10.1016/j.cej.2019.04.142.

[ref14] Fu Y.-F.; Yi F.-L.; Liu J.-R.; Li Y.-Q.; Wang Z.-Y.; Yang G.; Huang P.; Hu N.; Fu S.-Y. Super Soft but Strong E-Skin Based on Carbon Fiber/Carbon Black/Silicone Composite: Truly Mimicking Tactile Sensing and Mechanical Behavior of Human Skin. Compos. Sci. Technol. 2020, 186, 107910 10.1016/j.compscitech.2019.107910.

[ref15] Ata S.; Kobashi K.; Yumura M.; Hata K. Mechanically Durable and Highly Conductive Elastomeric Composites from Long Single-Walled Carbon Nanotubes Mimicking the Chain Structure of Polymers. Nano Lett. 2012, 12 (6), 2710–2716. 10.1021/nl204221y. 22546049

[ref16] Zuruzi A. S.; Haffiz T. M.; Affidah D.; Amirul A.; Norfatriah A.; Nurmawati M. H. Towards Wearable Pressure Sensors Using Multiwall Carbon Nanotube/Polydimethylsiloxane Nanocomposite Foams. Materials & Design 2017, 132, 449–458. 10.1016/j.matdes.2017.06.059.

[ref17] Shi G.; Zhao Z.; Pai J.-H.; Lee I.; Zhang L.; Stevenson C.; Ishara K.; Zhang R.; Zhu H.; Ma J. Highly Sensitive, Wearable, Durable Strain Sensors and Stretchable Conductors Using Graphene/Silicon Rubber Composites. Adv. Funct. Mater. 2016, 26 (42), 7614–7625. 10.1002/adfm.201602619.

[ref18] Chen Z.; Xi J.; Huang W.; Yuen M. M. F. Stretchable Conductive Elastomer for Wireless Wearable Communication Applications. Sci. Rep. 2017, 7 (1), 10958 10.1038/s41598-017-11392-w. 28887503 PMC5591259

[ref19] Larmagnac A.; Eggenberger S.; Janossy H.; Vörös J. Stretchable Electronics Based on Ag-PDMS Composites. Sci. Rep. 2014, 4, 7254 10.1038/srep07254. 25434843 PMC4248267

[ref20] Chen J.; Yu Q.; Cui X.; Dong M.; Zhang J.; Wang C.; Fan J.; Zhu Y.; Guo Z. An Overview of Stretchable Strain Sensors from Conductive Polymer Nanocomposites. Journal of Materials Chemistry C 2019, 7 (38), 11710–11730. 10.1039/C9TC03655E.

[ref21] Zheng Y.; Li Y.; Li Z.; Wang Y.; Dai K.; Zheng G.; Liu C.; Shen C. The Effect of Filler Dimensionality on the Electromechanical Performance of Polydimethylsiloxane Based Conductive Nanocomposites for Flexible Strain Sensors. Compos. Sci. Technol. 2017, 139, 64–73. 10.1016/j.compscitech.2016.12.014.

[ref22] Yang H.; Yao X.; Zheng Z.; Gong L.; Yuan L.; Yuan Y.; Liu Y. Highly Sensitive and Stretchable Graphene-Silicone Rubber Composites for Strain Sensing. Compos. Sci. Technol. 2018, 167, 371–378. 10.1016/j.compscitech.2018.08.022.

[ref23] Zhu S.; So J. H.; Mays R.; Desai S.; Barnes W. R.; Pourdeyhimi B.; Dickey M. D. Ultrastretchable Fibers with Metallic Conductivity Using a Liquid Metal Alloy Core. Adv. Funct. Mater. 2013, 23 (18), 2308–2314. 10.1002/adfm.201202405.

[ref24] Park J.; You I.; Shin S.; Jeong U. Material Approaches to Stretchable Strain Sensors. ChemPhysChem 2015, 16 (6), 1155–1163. 10.1002/cphc.201402810. 25641620

[ref25] Wang L.; Chiang W. H.; Loh K. J. Topological Design of Strain Sensing Nanocomposites. Sci. Rep. 2022, 12 (1), 9179 10.1038/s41598-022-13393-w. 35654931 PMC9163329

[ref26] Huang H.; Cai C. J.; Yeow B. S.; Ouyang J.; Ren H. Highly Stretchable and Kirigami-Structured Strain Sensors with Long Silver Nanowires of High Aspect Ratio. Machines 2021, 9 (9), 186 10.3390/machines9090186.

[ref27] Yong K.; De S.; Hsieh E. Y.; Leem J.; Aluru N. R.; Nam S. Kirigami-Inspired Strain-Insensitive Sensors Based on Atomically-Thin Materials. Mater. Today 2020, 34, 58–65. 10.1016/j.mattod.2019.08.013.

[ref28] Xu F.; Zhu Y. Highly Conductive and Stretchable Silver Nanowire Conductors. Advanced materials 2012, 24 (37), 5117–5122. 10.1002/adma.201201886. 22786752

[ref29] Catenacci M. J.; Reyes C.; Cruz M. A.; Wiley B. J. Stretchable Conductive Composites from Cu–Ag Nanowire Felt. ACS Nano 2018, 12 (4), 3689–3698. 10.1021/acsnano.8b00887. 29537819

[ref30] Yao S.; Zhu Y. Nanomaterial-Enabled Stretchable Conductors: Strategies Materials and Devices. Advanced materials 2015, 27 (9), 1480–1511. 10.1002/adma.201404446. 25619358

[ref31] Trung T. Q.; Lee N. E. Recent Progress on Stretchable Electronic Devices with Intrinsically Stretchable Components. Adv. Mater. 2017, 29 (3), 1603167 10.1002/adma.201603167. 27862355

[ref32] Yun G.; Tang S.-Y.; Lu H.; Zhang S.; Dickey M. D.; Li W. Hybrid-Filler Stretchable Conductive Composites: from Fabrication to Application. Small Sci. 2021, 1 (6), 2000080 10.1002/smsc.202000080.

[ref33] Cai G.; Wang J.; Qian K.; Chen J.; Li S.; Lee P. S. Extremely Stretchable Strain Sensors Based on Conductive Self-Healing Dynamic Cross-Links Hydrogels for Human-Motion Detection. Adv. Sci. 2017, 4 (2), 1600190 10.1002/advs.201600190. PMC5323873 28251045

[ref34] Lee W.; Kim H.; Kang I.; Park H.; Jung J.; Lee H.; Park H.; Park J. S.; Yuk J. M.; Ryu S. Universal Assembly of Liquid Metal Particles in Polymers Enables Elastic Printed circuit board. Science 2022, 378 (6620), 637–641. 10.1126/science.abo6631. 36356149

[ref35] Liu S.; Shah D. S.; Kramer-Bottiglio R. Highly Stretchable Multilayer Electronic Circuits Using Biphasic Gallium-Indium. Nat. Mater. 2021, 20 (6), 851–858. 10.1038/s41563-021-00921-8. 33603186

[ref36] Markvicka E. J.; Bartlett M. D.; Huang X.; Majidi C. An Autonomously Electrically Self-Healing Liquid Metal–Elastomer Composite for Robust Soft-Matter Robotics and Electronics. Nature materials 2018, 17 (7), 618–624. 10.1038/s41563-018-0084-7. 29784995

[ref37] Tutika R.; Haque A. T.; Bartlett M. D. Self-Healing Liquid Metal Composite for Reconfigurable and Recyclable Soft Electronics. Commun. Mater. 2021, 2 (1), 64 10.1038/s43246-021-00169-4.

[ref38] Yuan J.; Zhang Y.; Li G.; Liu S.; Zhu R. Printable and Stretchable Conductive Elastomers for Monitoring Dynamic Strain with High Fidelity. Adv. Funct. Mater. 2022, 32 (34), 2204878 10.1002/adfm.202204878.

[ref39] Ohm Y.; Pan C.; Ford M. J.; Huang X.; Liao J.; Majidi C. An Electrically Conductive Silver–Polyacrylamide–Alginate Hydrogel Composite for Soft Electronics. Nature Electronics 2021, 4 (3), 185–192. 10.1038/s41928-021-00545-5.

[ref40] Wang T.; Liu Q.; Liu H.; Xu B.; Xu H. Printable and Highly Stretchable Viscoelastic Conductors with Kinematically Reconstructed Conductive Pathways. Adv. Mater. 2022, 34 (28), 2202418 10.1002/adma.202202418. 35523721

[ref41] Araby S.; Meng Q.; Zhang L.; Zaman I.; Majewski P.; Ma J. Elastomeric Composites Based on Carbon Nanomaterials. Nanotechnology 2015, 26 (11), 112001 10.1088/0957-4484/26/11/112001. 25705981

[ref42] Singh M.; Vander Wal R. L. Nanostructure Quantification of Carbon Blacks. C 2018, 5 (1), 2 10.3390/c5010002.

[ref43] Sheka E.; Golubev Y. A.; Popova N. Raman Scattering by sp $^ 2$ Amorphous Carbons, condensed matter. arXiv preprint arXiv:2002.09913 2020, 10.48550/arXiv.2002.09913.

[ref44] Khodabakhshi S.; Fulvio P. F.; Andreoli E. Carbon Black Reborn: Structure and Chemistry for Renewable Energy Harnessing. Carbon 2020, 162, 604–649. 10.1016/j.carbon.2020.02.058.

[ref45] Ram R.; Rahaman M.; Aldalbahi A.; Khastgir D. Determination of Percolation Threshold and Electrical Conductivity of Polyvinylidene Fluoride (PVDF)/Short Carbon Fiber (SCF) Composites: Effect of SCF Aspect Ratio. Polym. Int. 2017, 66 (4), 573–582. 10.1002/pi.5294.

[ref46] Neffati R.; Brokken-Zijp J. Electric Conductivity in Silicone-Carbon Black Nanocomposites: Percolation and Variable Range Hopping on a Fractal. Materials Research Express 2019, 6 (12), 125058 10.1088/2053-1591/ab58fd.

[ref47] Huang Y.; Kormakov S.; He X.; Gao X.; Zheng X.; Liu Y.; Sun J.; Wu D. Conductive Polymer Composites from Renewable Resources: an Overview of Preparation, Properties, and Applications. Polymers 2019, 11 (2), 187 10.3390/polym11020187. 30960171 PMC6418900

[ref48] Gabbett C.; Kelly A. G.; Coleman E.; Doolan L.; Carey T.; Synnatschke K.; Liu S.; Dawson A.; O’Suilleabhain D.; Munuera J. Understanding how Junction Resistances Impact the Conduction Mechanism in Nano-Networks. Nat. Commun. 2024, 15 (1), 4517 10.1038/s41467-024-48614-5. 38806479 PMC11133347

[ref49] Bozó É.; Ervasti H.; Halonen N.; Shokouh S. H. H.; Tolvanen J.; Pitkanen O.; Jarvinen T.; Palvolgyi P. S.; Szamosvolgyi A.; Sápi A. Bioplastics and Carbon-Based Sustainable Materials, Components, and Devices: Toward Green Electronics. ACS Appl. Mater. Interfaces 2021, 13 (41), 49301–49312. 10.1021/acsami.1c13787. 34609829 PMC8532127

[ref50] Coupette F.; Zhang L.; Kuttich B.; Chumakov A.; Roth S. V.; González-García L.; Kraus T.; Schilling T. Percolation of Rigid Fractal Carbon Black Aggregates. J. Chem. Phys. 2021, 155 (12), 124902 10.1063/5.0058503. 34598569

[ref51] Gao Q.; Liu J.; Liu X. Electrical Conductivity and Rheological Properties of Carbon Black Based Conductive Polymer Composites Prior To and After Annealing. Polym. Polym. Compos. 2021, 29 (9_suppl), S288–S295. 10.1177/09673911211001277.

[ref52] Du J.; Cheng H. M. The Fabrication, Properties, and Uses of Graphene/Polymer Composites. Macromol. Chem. Phys. 2012, 213 (10–11), 1060–1077. 10.1002/macp.201200029.

[ref53] Rwei S.-P.; Ku F.-H.; Cheng K.-C. Dispersion of Carbon Black in a Continuous Phase: Electrical, Rheological, and Morphological studies. Colloid Polym. Sci. 2002, 280 (12), 1110–1115. 10.1007/s00396-002-0718-8.

[ref54] Bauhofer W.; Kovacs J. Z. A Review and Analysis of Electrical Percolation in Carbon Nanotube Polymer Composites. Compos. Sci. Technol. 2009, 69 (10), 1486–1498. 10.1016/j.compscitech.2008.06.018.

[ref55] Subramanian S.; Øye G. Aqueous Carbon Black Dispersions Stabilized by Sodium Lignosulfonates. Colloid Polym. Sci. 2021, 299, 1223–1236. 10.1007/s00396-021-04840-7.

[ref56] Penu C.; Hu G. H.; Fernandez A.; Marchal P.; Choplin L. Rheological and Electrical Percolation Thresholds of Carbon Nanotube/Polymer Nanocomposites. Polym. Eng. Sci. 2012, 52 (10), 2173–2181. 10.1002/pen.23162.

[ref57] Mezger T. G. Das Rheologie Handbuch; Vincentz Network, 2016.

[ref58] Kreyenschulte H.; Richter S.; Götze T.; Fischer D.; Steinhauser D.; Klüppel M.; Heinrich G. Interaction of 1-Allyl-3-Methyl-Imidazolium Chloride and Carbon Black and Its Influence on Carbon Black Filled Rubbers. Carbon 2012, 50 (10), 3649–3658. 10.1016/j.carbon.2012.03.037.

[ref59] Chung D. A Critical Review of Piezoresistivity and Its Application in Electrical-Resistance-Based Strain Sensing. J. Mater. Sci. 2020, 55 (32), 15367–15396. 10.1007/s10853-020-05099-z.

