# Supplementary material for: Electrofluids with Tailored Rheoelectrical Properties: Liquid Composites with Tunable Network Structures as Stretchable Conductors
Source: ACS Appl Mater Interfaces. 2024 Aug 8;16(33):43942–50. doi: 10.1021/acsami.4c07230 (PMC11345724; doi:10.1021/acsami.4c07230)
Supplement: Supplementary file 1 — am4c07230_si_001.pdf [file am4c07230_si_001.pdf]

SUPPORTING INFORMATION

Electrofluids with Tailored Rheoelectrical Properties: Liquid Composites with Tunable Network Structures as Stretchable Conductors

Dominik S. Schmidt[1], *Tobias Kraus*[1,2], *Lola González-García*[1,3]*.

[1]INM-Leibniz Institute for New Materials, Campus D2 2, 66123 Saarbrücken, Germany

[2]Saarland University, Colloid and Interface Chemistry, Campus D2 2, 66123 Saarbrücken, Germany

[3]Saarland University, Department of Materials Science and Engineering, Campus D2 2, 66123 Saarbrücken, Germany

*Lola.gonzalez-garcia@leibniz-inm.de

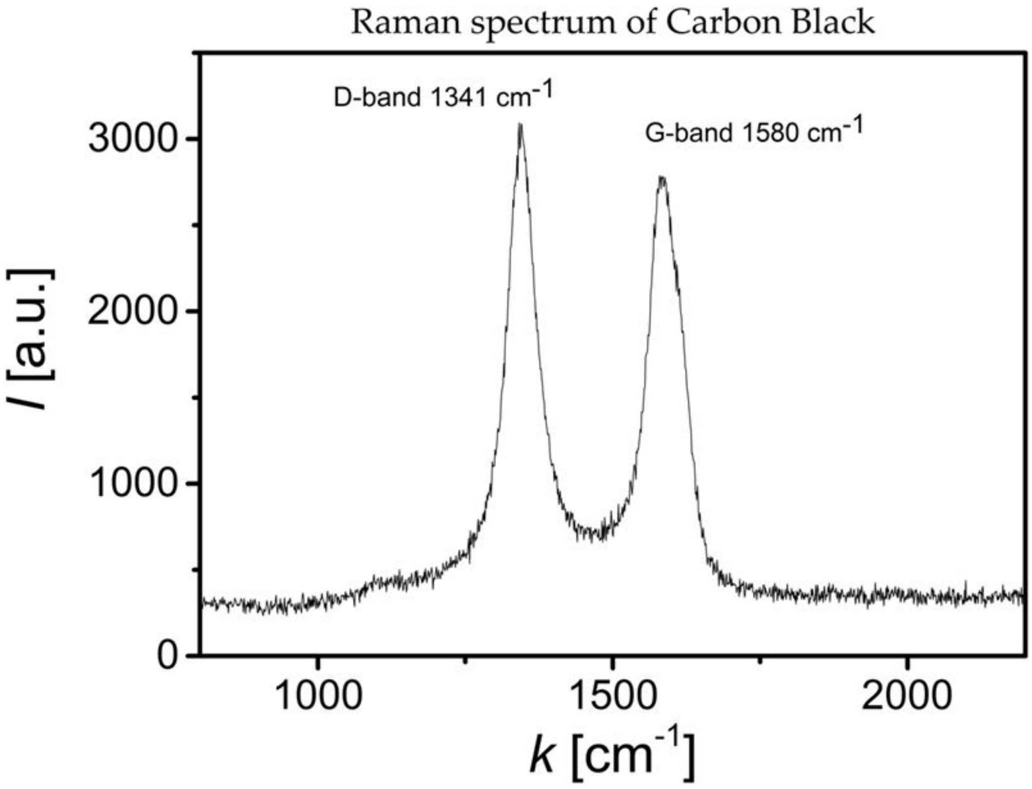

Figure S1: Raman spectrum of Carbon Black powder.

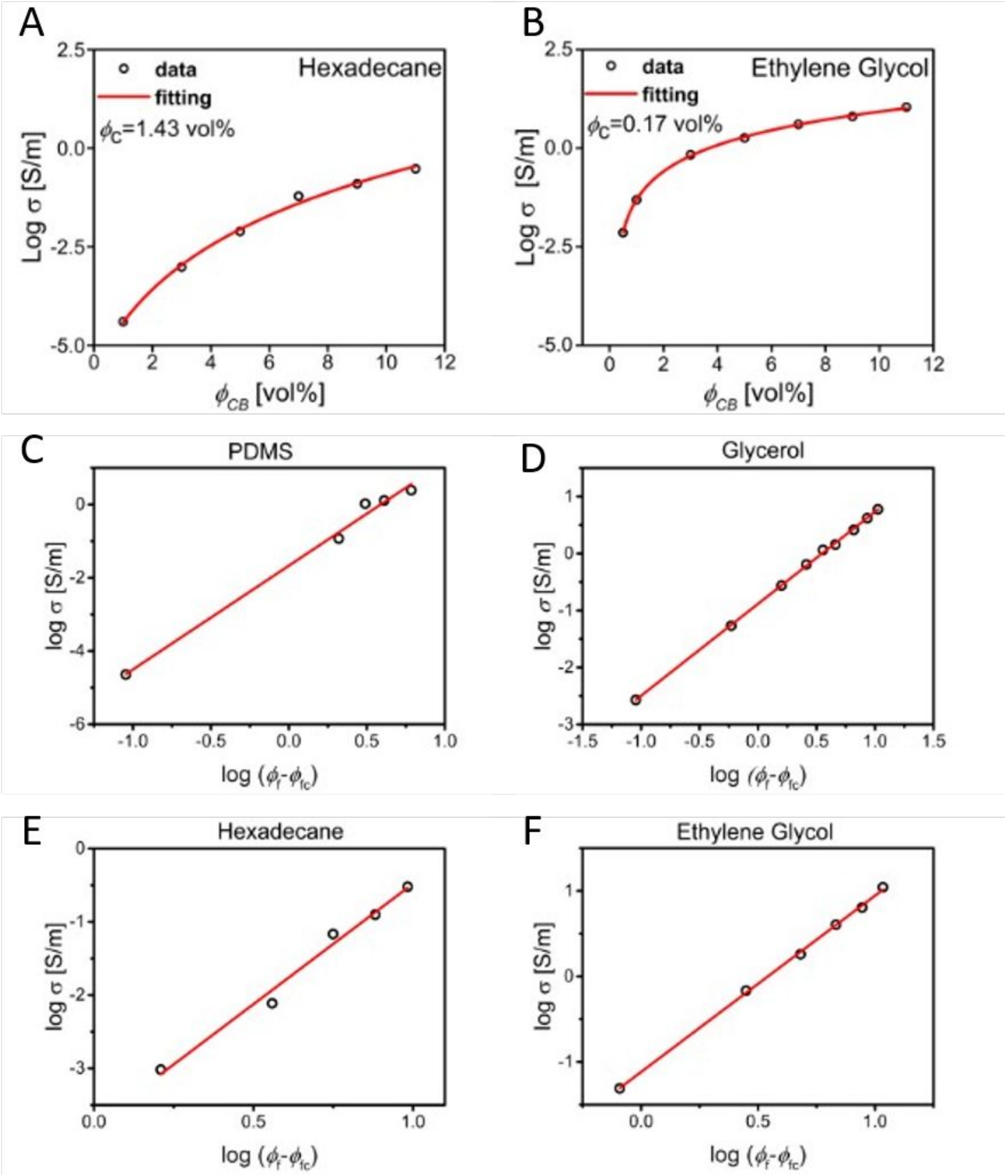

Figure S2: Percolation threshold for CB in hexadecane (A) and ethylene glycol (B). Linear fitting curves of the percolation threshold determination for the CB mixtures in PDMS (C), in glycerol (D), in hexadecane (E), and in ethylene glycol (F).

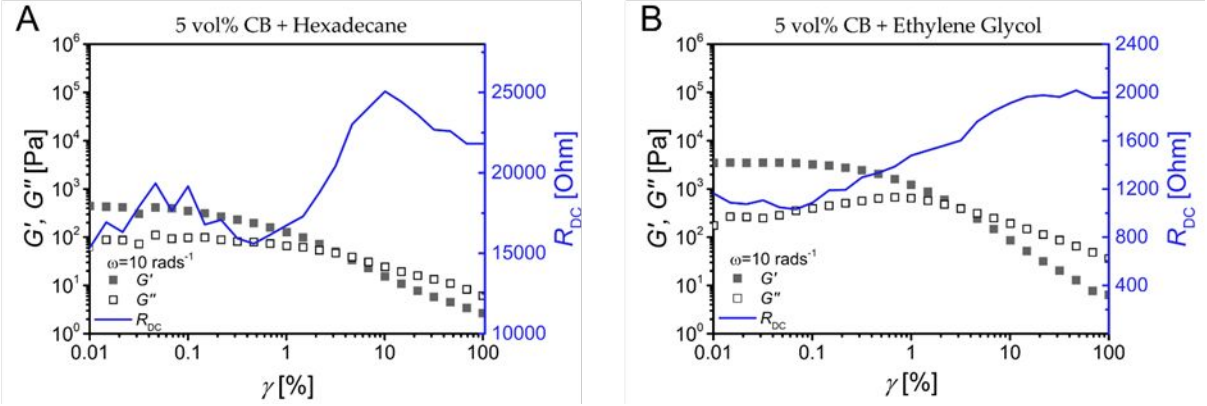

Figure S3: Rheoelectrical measurements of 5 vol% CB in A) hexadecane and B) ethylene glycol.

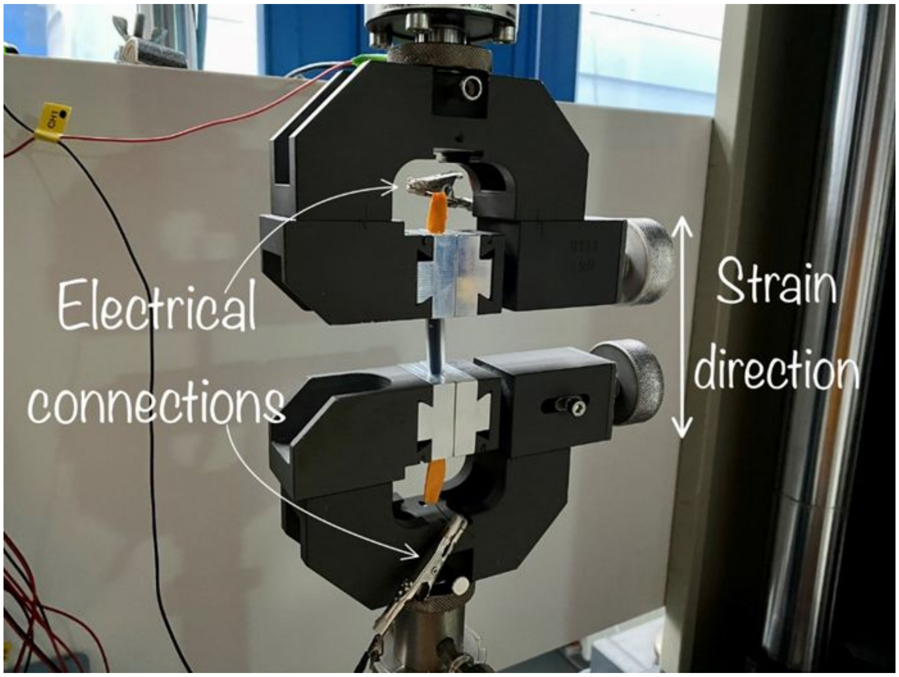

Figure S4: Image of the uniaxial tensile test set-up. The electrofluid was encapsulated in an EcoFlex® tube, clamped into the tensile machine, and electrically connected to the Keithley DAQ6510 Sourcemeter.

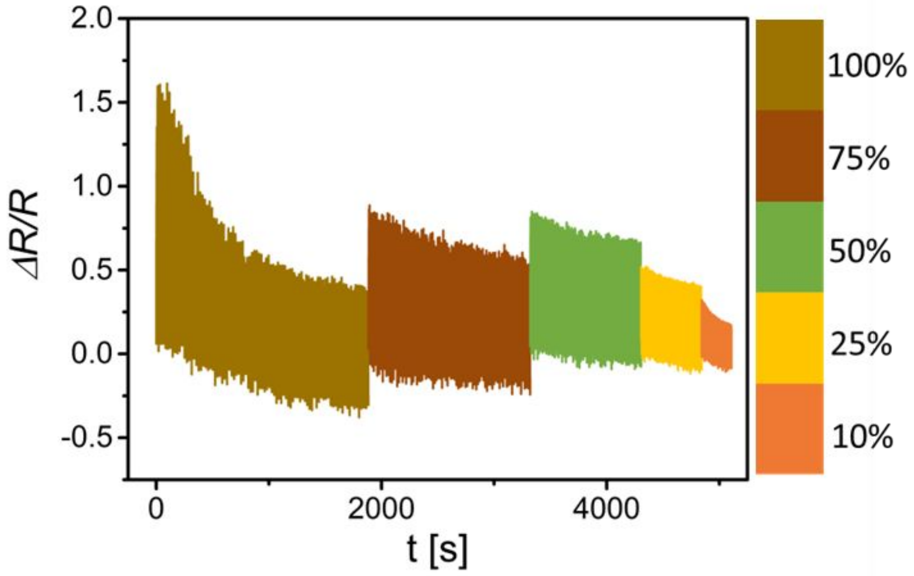

Figure S5: Change in resistance of a 9 vol% CB in PDMS sample, stretched to different strain amplitudes. From brown to orange 100%, 75%, 50%, 25%, and 10%. Each strain value was cycled for 100 cycles.

Videos of the demonstrators can be found as electronic supporting information: Video1 and Video2.
